# Participation of TDP1 in the repair of formaldehyde-induced DNA-protein cross-links in chicken DT40 cells

**DOI:** 10.1371/journal.pone.0234859

**Published:** 2020-06-26

**Authors:** Toshiaki Nakano, Mahmoud I. Shoulkamy, Masataka Tsuda, Hiroyuki Sasanuma, Kouji Hirota, Minoru Takata, Shin-ichiro Masunaga, Shunichi Takeda, Hiroshi Ide, Tadayoshi Bessho, Keizo Tano

**Affiliations:** 1 DNA Damage Chemistry Research Group, Institute for Quantum Life Science, Kansai Photon Science Institute, National Institutes of Quantum and Radiological Science and Technology, Kizugawa, Kyoto, Japan; 2 Department of Mathematical and Life Sciences, Graduate School of Science, Hiroshima University, Higashi-Hiroshima, Japan; 3 Department of Radiation Genetics, Graduate School of Medicine, Kyoto University, Kyoto, Japan; 4 Department of Chemistry, Graduate School of Science, Tokyo Metropolitan University, Hachioji, Tokyo, Japan; 5 Department of Late Effects Studies, Radiation Biology Center, Kyoto University, Kyoto, Japan; 6 Division of Radiation Life Science, Institute for Integrated Radiation and Nuclear Science, Kyoto University, Kumatori, Osaka, Japan; 7 The Eppley Institute for Research in Cancer and Allied Diseases, Fred & Pamela Buffett Cancer Center, University of Nebraska Medical Center, Omaha, Nebraska, United States of America; University of South Alabama Mitchell Cancer Institute, UNITED STATES

## Abstract

Proteins are covalently trapped on DNA to form DNA-protein cross-links (DPCs) when cells are exposed to DNA-damaging agents. Aldehyde compounds produce common types of DPCs that contain proteins in an undisrupted DNA strand. Tyrosyl-DNA phosphodiesterase 1 (TDP1) repairs topoisomerase 1 (TOPO1) that is trapped at the 3’-end of DNA. In the present study, we examined the contribution of TDP1 to the repair of formaldehyde-induced DPCs using a reverse genetic strategy with chicken DT40 cells. The results obtained showed that cells deficient in TDP1 were sensitive to formaldehyde. The removal of formaldehyde-induced DPCs was slower in tdp1-deficient cells than in wild type cells. We also found that formaldehyde did not produce trapped TOPO1, indicating that trapped TOPO1 was not a primary cytotoxic DNA lesion that was generated by formaldehyde and repaired by TDP1. The formaldehyde treatment resulted in the accumulation of chromosomal breakages that were more prominent in tdp1-deficient cells than in wild type cells. Therefore, TDP1 plays a critical role in the repair of formaldehyde-induced DPCs that are distinct from trapped TOPO1.

## Introduction

Proteins can be covalently cross-linked to DNA by endogenous and exogenous agents and form DNA-protein cross-links (DPCs) [1, 2]. DPCs are caused by covalently linking DNA and DNA-associated proteins and by trapping the reaction intermediates of specific DNA-metabolizing enzymes. Examples of the former are DPCs containing histones and of the latter are DPCs containing topoisomerases (TOPOs), DNA polymerases, and DNA methyltransferases (DNMTs) [3–6]. Due to the large sizes of cross-linked proteins, DPCs inhibit various DNA transactions, such as DNA replication, transcription, and DNA repair [2]. Therefore, DPCs are highly cytotoxic. Several DNA repair mechanisms have been shown to process DPCs and maintain genome integrity [7]. When a DNA polymerase or replicative helicase is blocked by DPCs, DPCs can be repaired by SPRTN-mediated protein degradation followed by translesion DNA synthesis (TLS) and/or nucleotide excision repair (NER) [8–12]. Since SPRTN-mediated proteolysis targets various cross-linked proteins, it is regarded as the major DPC-repair pathway [13]. In contrast, DPCs containing trapped topoisomerases are repaired by a specialized pathway. Tyrosyl-DNA phosphodiesterase 1 (TDP1), and TDP2 remove trapped TOPO1 and TOPO2, respectively [14, 15]. Trapped TOPO1 at the 3’-end of a single-strand break (SSB) is subjected to proteasomal degradation, and TDP1 removes the resulting cross-linked proteins. The resulting SSB is repaired by the general SSB repair pathway. Proteasomal degradation breaks down trapped TOPO2s at the 5’-ends of a double-strand break (DSB) and TDP2 removes the degraded proteins. Homologous recombination (HR) or non-homologous end-joining (NHEJ) then fixes the DSB. Trapped TOPO2 is also repaired through the MRE11-RAD50-NBS1 (MRN) complex-mediated pathway [16–19].

Formaldehyde is produced during normal cell metabolism. The enzymatic demethylation of histones, DNA and RNA, and AlkB-dependent DNA repair produce formaldehyde in nuclei [20–23]. Formaldehyde generates various types of DNA damage, such as DNA intra-strand cross-links, DNA inter-strand cross-links (ICLs), base modifications, and DPCs [1, 24–27]. These DNA lesions contribute to formaldehyde-induced cytotoxicity, mutagenicity, and carcinogenicity. Therefore, several DNA repair pathways are responsible for the repair of formaldehyde-induced DNA lesions. The Fanconi anemia pathway promotes the repair of ICLs and the mechanism of the Fanconi anemia pathway has been studied extensively [28]. Since cells defective in the Fanconi anemia pathway are hypersensitive to formaldehyde as well as DNA cross-linking agents, Fanconi anemia-mediated ICL repair activity has also been suggested to be required for the repair of formaldehyde-induced DPCs [29–33]. Formaldehyde concurrently induces ICLs and DPCs; however, it is technically difficult to assess their relative yields. Furthermore, previous studies indicated no role for FANCD2 in the repair of DPCs [8, 11, 12]. Thus, the role of the Fanconi anemia pathway in the repair of formaldehyde-induced DPCs remains unclear.

TOPOs have nicking and relegation activities to relieve the tension of DNA strands [34]. Camptothecin (CPT) and etoposide (VP16) stabilize the reaction intermediates of TOPO1 and TOPO2 moiety, respectively, and produce covalently trapped TOPOs at the DNA end (S1 Fig). TDP1 and TDP2 release trapped TOPO1 and TOPO2 residues from DNA ends [35]. Thus, tdp1- and tdp2-deficient cells display hypersensitivity to CPT or VP16, respectively [35]. Increasing evidence demonstrates that TDP1 has the capacity to remove a broad range of 3’-end blocking DNA lesions (3’-cleavage activity) [36–39]. Recent studies also reported that tdp1-deficient cells were sensitive to VP16 that trapped TOPO2-DNA reaction intermediates [38–43]. Thus, TDP1 removes trapped TOPO2 at 5’-end of DSBs.

In the present study, we investigated the roles of TDP1 and TDP2 in the repair of formaldehyde-induced DNA damage in DT40 cell lines. Due to high gene targeting efficiency, DT40 cells have been widely used to evaluate the genotoxicity of a number of chemical compounds [44]. Since experiments can be performed using various mutant cell lines that are isogenic to the wild type, the data obtained are interpreted without a genetic bias. We herein demonstrated that tdp1-deficient DT40 cells were sensitive to formaldehyde, whereas tdp2-deficient DT40 cells were not. Furthermore, tdp1-deficient cells were not sensitive to mitomycin C (MMC) that selectively induces ICLs, strongly suggesting that DPCs, but not ICLs, are the cytotoxic lesions generated by formaldehyde. Biochemical experiments showed that the removal of formaldehyde-induced DPCs was slower in tdp1-deficient cells than in wild type cells. However, trapped TOPO1 was not observed in wild type or tdp1-deficient cells, showing that trapped TOPO1 was not a primary DNA lesion that was generated by formaldehyde and repaired by TDP1. Formaldehyde-induced DPCs resulted in chromosomal breakages that were more prominent in tdp1-deficient cells than in wild type cells. Therefore, TDP1 participates in the repair of formaldehyde-induced DPCs that are distinct from trapped TOPO1. We propose that TDP1 removes DPCs in addition to trapped TOPO1, the canonical substrate in cells.

## Materials and methods

### Cell lines and cell culture

The DT40 cell lines used in the present study were generated in the Laboratory of Radiation Genetics, Graduate School of Medicine, Kyoto University or Late Effects Studies Laboratory of DNA damage signaling in the Radiation Research Center, Kyoto University (Kyoto Japan). All DT40 mutants used were previously authenticated by Southern blotting, PCR, and/or Western blotting and are isogenic to the wild type cell line (S1 Table). Wild type and mutant DT40 cells were cultured at 39°C with 5% CO_2_ in RPMI 1640 medium supplemented with 10% fetal bovine serum, 1% chicken serum, and 2 mM L–glutamine [45–47].

### Measurement of cytotoxicity

A colony formation assay was performed to evaluate the sensitivity of DNA repair-deficient DT40 mutants to drugs [45–47]. Briefly, serially diluted cells were plated in triplicated 60-mm dishes with 8 ml of DMEM/F-12 containing 1.5% methylcellulose, 2 mM L-glutamine, 15% of fetal calf serum, and 1.5% of chicken serum. Cells were incubated in complete RPMI 1640 medium with or without the indicated concentrations of the drug. After a 3-hour incubation with formaldehyde or 24-hour incubation with MMC, serially diluted cells were plated in triplicated dishes containing methylcellulose and DMEM/F112 medium. Colonies were counted after seven days of incubation at 39°C.

### Detection of DPCs by fluorescent labeling

DPCs were detected by the previously established method with some modifications [26, 48]. Briefly, DT40 cells were treated with 0.2 mM formaldehyde for 3 hours. After washing formaldehyde-treated cells, the cells were further incubated with fresh media for the indicated times. Cells were collected and lysed in 10% Sarkosyl solution, and chromosomal DNA was purified by two cycles of CsCl density gradient centrifugation. Purified chromosomal DNA was incubated with 0.1 mM FITC that exclusively reacts with amino groups in proteins. Then, the amount of DPCs were quantified by fluorometry.

### Immunodetection of DPCs containing TOPO1 and histone H3

To detect TOPO1 and histone H3 covalently cross-linked to DNA after the formaldehyde treatment, chromosomal DNA was isolated and purified by ultra-centrifugation. Purified DNA (30 μg) was digested with DNase I at 37°C and the resulting products were separated through a 4–12% (w/v) bis-Tris SDS polyacrylamide gel (Bio-Rad). The separated products were electro-blotted onto a membrane and detected with anti-topoisomerase 1 (Bethyl Laboratories, cat# 302-590A) or anti-histone H3 antibodies, followed by horseradish peroxidase-conjugated secondary antibodies (GE Healthcare) and ECL or ECL Prime Chemiluminescence reagent (Bio-Rad).

Trapped TOPO1 was also detected by slot blot analysis. Chromosomal DNA was purified as described above and purified DNA (30 μg) was transferred to nitrocellulose membrane by vacuum with a slot blot apparatus. After washing with TBST (20 mM Tris HCl pH7.6, 137 mM NaCl and 0.1% Tween 20), the membrane was incubated with anti-topoisomerase 1 antibody, followed by horseradish peroxidase-conjugated secondary antibodies (GE Healthcare) and ECL or ECL Prime Chemiluminescence reagent (Bio-Rad). Purified TOPO1 was used as positive control.

### Chromosome aberration assays

Chromosomal aberrations were detected as described previously [45–47]. Briefly, formaldehyde-treated cells were treated for three hours with medium containing 0.1 μg/ml colcemid (Gibco). Harvested cells were incubated in 1 ml of 75 mM KCl at room temperature for 15 minutes and fixed in 5 ml of a freshly prepared 3:1 mixture of methanol-acetic acid. The cell suspension was dropped onto a slide, and the slides were dried. The slides were stained with 5% Giemsa solution (pH 6.4) for 8 minutes. We focused on the generation of chromatid and iso-chromatid breaks. Data are presented as macro chromosomal aberrations per 20 meta-phase spreads.

### Statistical analysis

Three independent experiments were performed with each data set in the present study unless stated otherwise. Data were expressed as the means ± S.D. unless stated otherwise. The significance of differences was examined using the Student’s *t*-test and *p*-values<0.05 were considered to be significant.

## Results and discussion

### *Tdp1* cells are sensitive to formaldehyde

**Fig 1:**
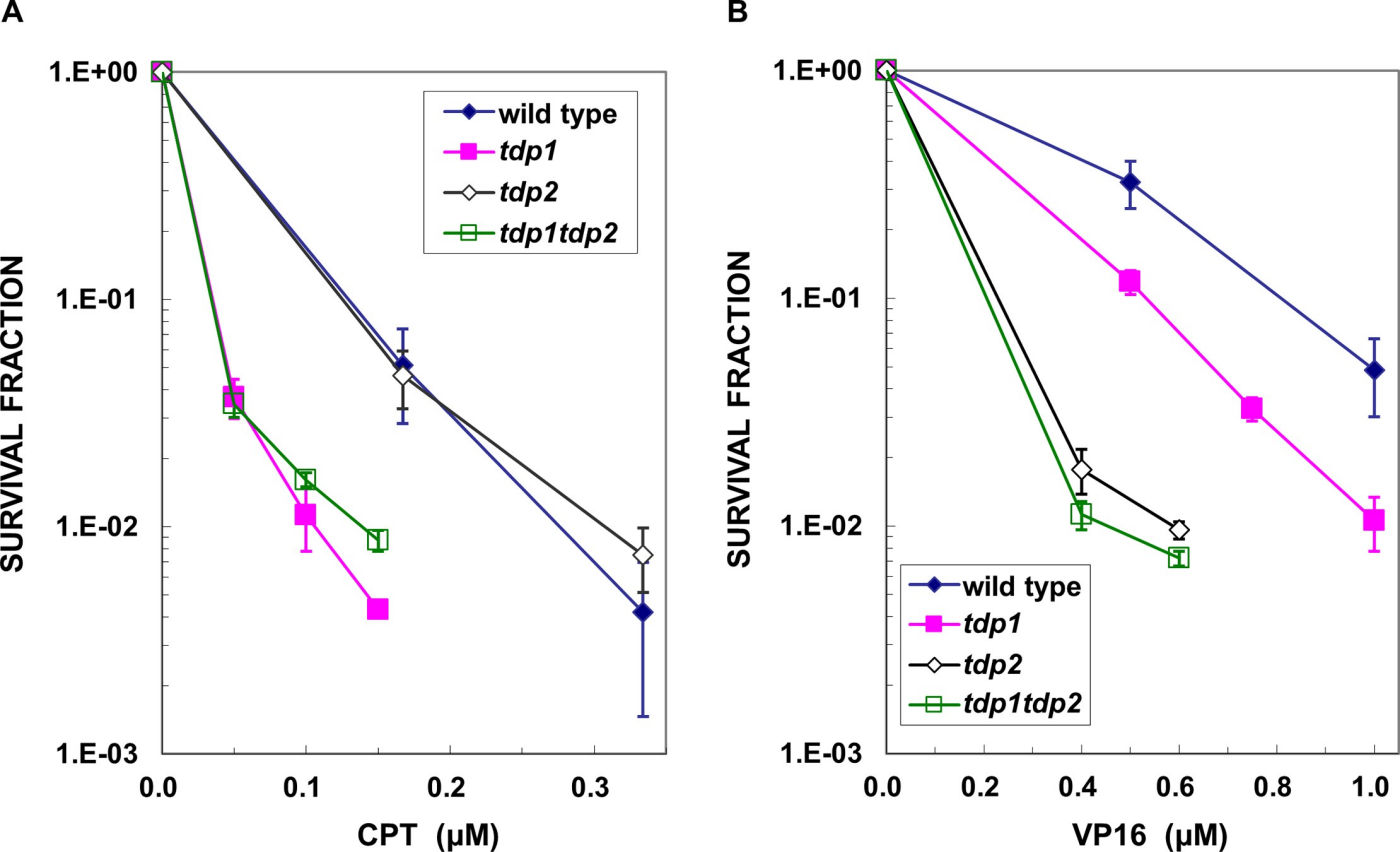
Cellular sensitivities of DT40 cells deficient in TDP1 (*tdp1*), TDP2 (*tdp2*), and both TDP1 and TDP2 (*tdp1tdp2*) to the topoisomerase 1 inhibitor, camptothecin (CPT) and the topoisomerase 2 inhibitor, etoposide (VP16). (A) CPT was highly cytotoxic to *tdp1*, but not *tdp2* cells; (B) *Tdp2* cells were hypersensitive to VP16 while *tdp1* cells were moderately sensitive to VP16. All data represent the mean ± SD from at least three independent experiments.

To examine whether TDP1 and TDP2 are involved in the repair of DPCs with proteins that are covalently attached to the undisrupted DNA strand, we generated tdp1- and tdp2-deficient DT40 cells. Cells deficient in TDP1 (*tdp1*) and TDP2 (*tdp2*) showed hypersensitivity to CTP (Fig 1A) and VP16 (Fig 1B), respectively. The 3’-cleavage activity of TDP1 has been shown to be involved in the removal of DNA damage induced by CPT and its derivatives, several nucleoside analogs and alkylating agents [36–38, 40, 41]. Consistent with previous findings [38, 40–43], *tdp1* DT40 cells were also sensitive to VP16 (Fig 1B). These results confirm the known cellular phenotypes of tdp1- and tdp2-deficient cells.

**Fig 2:**
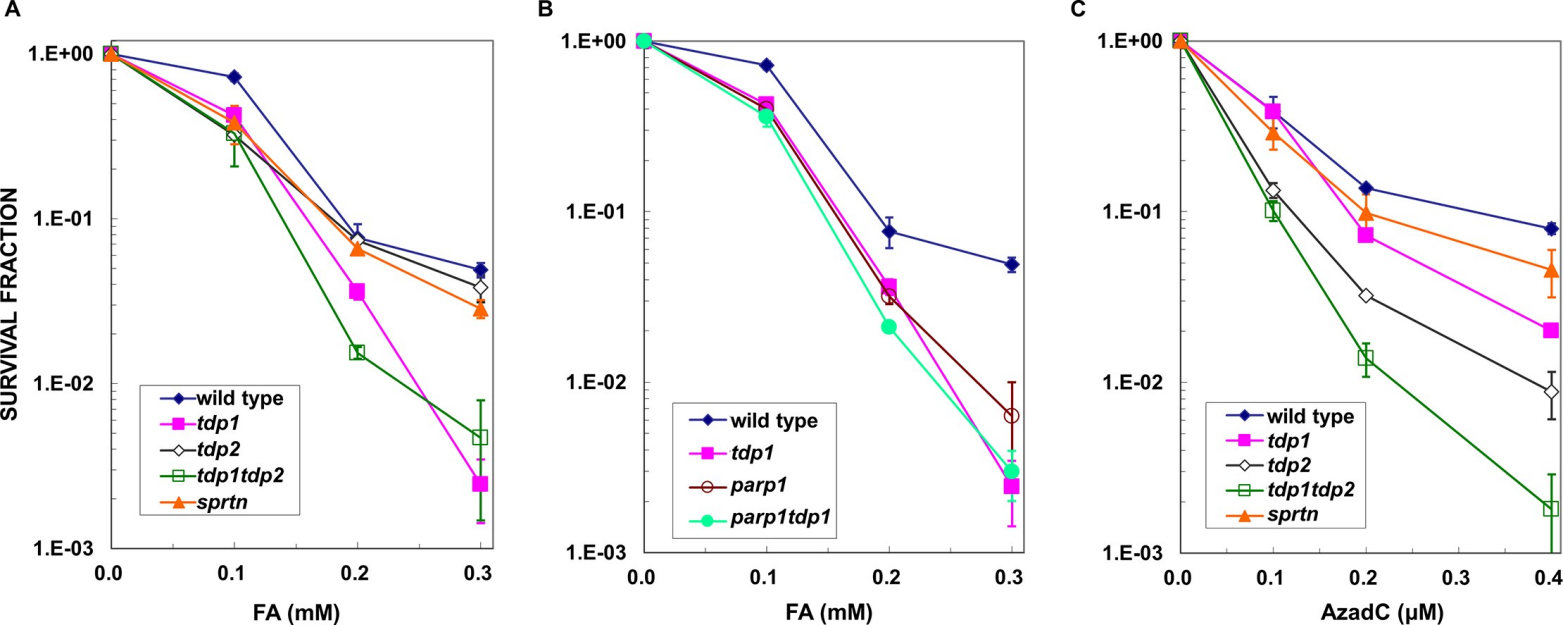
Cellular sensitivities of tdp1-, tdp2-, and sprtn-deficient cells to formaldehyde and 5-aza-2’-deoxycytidine (AzadC). (A) *Tdp1* cells were moderately sensitive to formaldehyde, while *tdp2* cells were not. Cells deficient in TDP1 and TDP2 (*tdp1tdp2*) exhibited similar formaldehyde sensitivity to *tdp1* cells. *Sprtn* cells were not sensitive to formaldehyde; (B) TDP1 and PARP1 were epistatic in cellular resistance to formaldehyde-induced cytotoxic lesions. Parp1-deficient (*parp1*) cells were sensitive to formaldehyde and *tdp1parp1* double mutant cells showed similar formaldehyde sensitivity to *tdp1* cells; (C) *Tdp1* and *tdp2* cells were sensitive to AzadC. *Tdp1* and *tdp2* cells were both sensitive to AzadC. *Tdp1tdp2* double mutant cells were more sensitive to AzadC than *tdp2* cells. *Sprtn* cells were weakly sensitive to AzadC. All data represent the mean ± SD of at least three independent experiments.

Next, we investigated whether TDP1 exhibits repair activity to aldehyde-induced DPCs. Aldehydes, particularly formaldehyde, are known to preferentially produce DPCs without disruption of DNA [2, 13, 25]. To examine whether TDP1 and TDP2 are involved in the repair of formaldehyde-induced DPCs, we treated wild type, *tdp1*, and *tdp2* cells with increasing concentrations of formaldehyde for 3 hours. Interestingly, *tdp1* cells were moderately sensitive to formaldehyde relative to wild type cells (Fig 2A). In contrast, tdp2-deficient cells did not show sensitivity to formaldehyde. Furthermore, *tdp1* mutant and *tdp1tdp2* double mutant cells showed similar sensitivity to formaldehyde (Fig 2A). These results strongly suggest that TDP1, but not TDP2, is uniquely involved in the repair of formaldehyde-induced DNA damage.

Formaldehyde generates various DNA adducts, such as base modifications, DNA intra-strand crosslinks, and ICLs, together with DPCs [1, 24, 25, 27]. Although there is no general method for the quantitation of the amount of ICLs in cells, ICLs are considered to be lethal DNA lesions produced by formaldehyde because cells derived from Fanconi anemia patients deficient in FANCC and FANCD2 are hypersensitive to formaldehyde [29]. DT40 cell lines deficient in the Fanconi anemia pathway-related genes showed hypersensitivity to both MMC (S3B and S3D Fig) and formaldehyde (S3C Fig). In contrast, *tdp1* cells did not show sensitivity to MMC that preferentially induces ICLs without forming DPCs (S3A Fig). These results indicate that ICLs are not substrates for TDP1. Thus, the cytotoxic effects of formaldehyde on *tdp1* cells were attributed to DPCs.

A previous study showed that poly(ADP-ribose)polymerase 1 (PARP1) bound the N-terminal domain of TDP1 and stimulated the excision of trapped TOPO1 by CPT and also that TDP1 and PARP1 are epistatic for CPT sensitivity [49]. Consistent with these findings, parp1-deficient cells were sensitive to formaldehyde and an additional disruption of the PARP1 gene in *tdp1* cells did not increase formaldehyde sensitivity (Fig 2B), showing an epistatic relationship between PARP1 and TDP1 in formaldehyde sensitivity. Thus, PARP1 may participate in TDP1-mediated DPC repair.

### Tdp1- and tdp2-deficient cells are sensitive to AzadC

The unexpected results with a contribution of TDP1 in the repair of formaldehyde-induced DPCs prompted us to investigate the role of TDP1 in removing DPCs induced by 5-Aza-2’-deoxycytidine (decitabine, AzadC). An anticancer drug, AzadC, is a nucleoside analog and incorporated randomly in the genome during DNA replication. The incorporated AzadC inhibits the activity of DNMT by forming a covalent bond between a C6 of AzadC in DNA and a thiol group of DNMT [1, 50, 51]. Thus, unlike DPCs that are randomly and non-specifically induced by formaldehyde, a treatment with AzadC results in DPCs containing a specific enzyme DNMT. In contrast to the formaldehyde treatment (Fig 2A), *tdp1* and *tdp2* cells were both hypersensitive to AzadC (Fig 2C). *Tdp2* cells showed higher sensitivity to AzadC than *tdp1* cells (Fig 2C). Furthermore, *tdp1tdp2* double mutant cells were more sensitive to AzadC than single *tdp1* or *tdp2* mutant cells (Fig 2C). Thus, TDP1 and TDP2 appear to participate in redundant pathways to remove the DPCs induced by AzadC.

AzadC induces RAD51 foci formation that is a marker for the HR process [52, 53]. Furthermore, DSBs accumulated in HR-deficient mammalian cells after a treatment with AzadC [52–54]. Interestingly, it was shown that, using a plasmid replication system in *E*. *coli*, AzadC-induced DPCs block DNA replication and generate RecA-dependent X-structures that can be intermediate structures of HR [55]. These findings suggest that AzadC-induced DPCs generate DSBs through collapsed DNA replication forks at or near the DPCs. An incision 5’-side of DPCs at a stalled replication fork may provide a DSB, a substrate for TDP2. TDP1 may act on the same substrate.

SPRTN is a key metalloprotease that degrades the proteins of DPCs in DNA replication-coupled DPC repair [8–12]. Since SPRTN degrades various DPCs, SPRTN-mediated DPC repair is considered to be versatile. Sprtn-deficient cells (*sprtn*) showed sensitivity to AzadC. However, sensitivity was markedly weaker than that in *tdp1* and *tdp2* cells (Fig 2C). It is also important to note that sprtn-deficient cells were not sensitive to formaldehyde (Fig 2A). The results are inconsistent with previous reports that showed a role of SPRTN in formaldehyde-induced DPCs in mammalian cells [10–12]. We do not know the reason for the discrepancy, however, a contribution of SPRTN in the repair of formaldehyde-induced DPCs might be different in DT40. High efficiency of HR in DT40 might contribute to no sensitivity to formaldehyde in the *sprtn* cells mutant DT40. In yeast, Wss1, a homolog of SPRTN, degrades DPCs and the Wss1-dependent proteolysis functions parallel to HR to counter DPCs [56, 57]. Thus, a highly efficient HR might be able to compensate the lack of SPNTN-dependent degradation of DPCs in DT40.

The results in Fig 2C suggest that the removal of large DNMT-DPC partially depends on proteolytic degradation by SPRTN, and also emphasize the critical role of TDP1 and TDP2 in the repair of AzadC-induced DPCs in chicken DT40 cells

### Removal of formaldehyde-induced DPCs is delayed in tdp1-deficient cells

**Fig 3:**
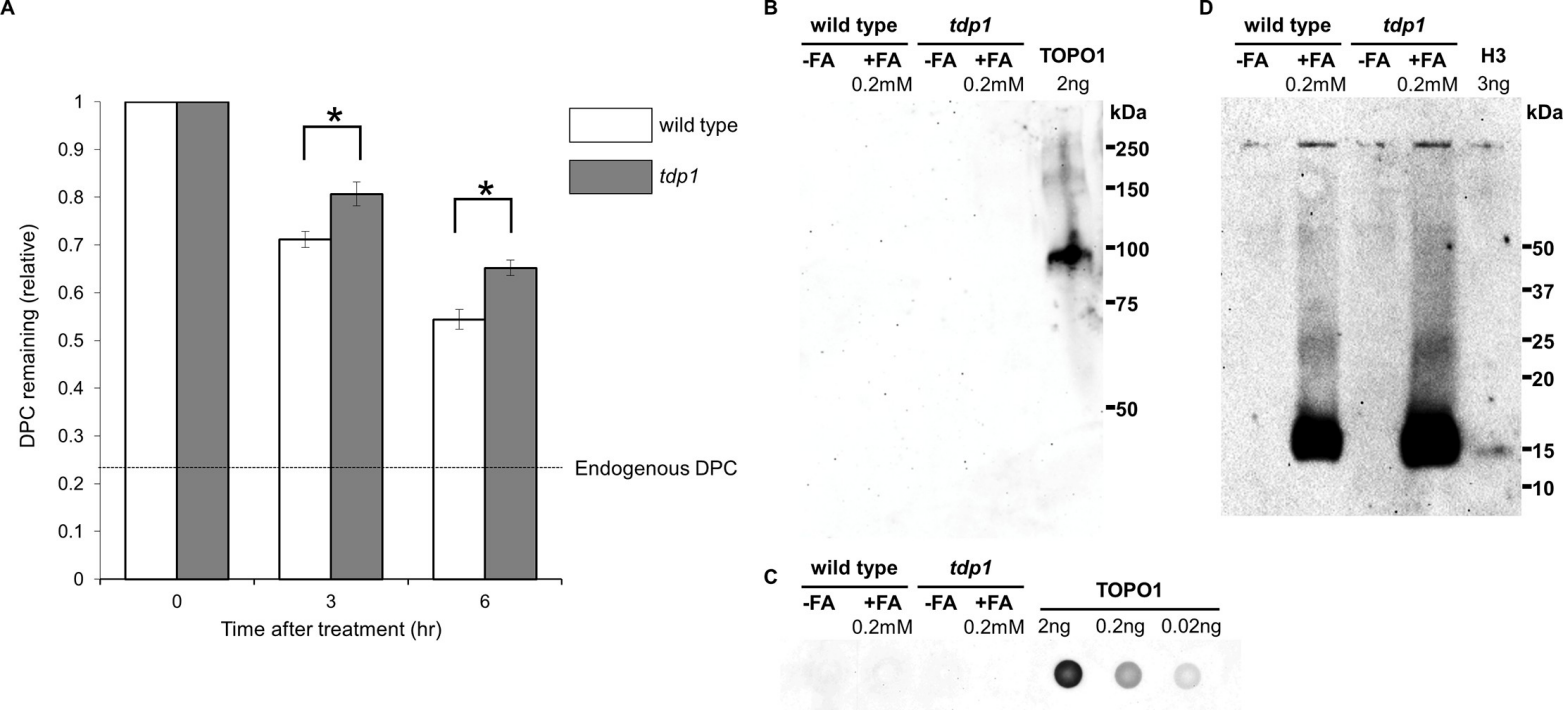
Repair of formaldehyde-induced DPCs is compromised in *tdp1* cells. (A) The removal of formaldehyde-induced DPCs was delayed in *tdp1* cells. The total amount of DPCs in the genome was measured by the FITC-fluorescence labeling method. Cells were treated with 0.2 mM formaldehyde for 3 hours. The relative amounts of DPCs after 3 hours were 0.712 ± 0.017 in wild type cells and 0.807 ± 0.025 in tdp1-deficient cells, and after 6 hours were 0.544 ± 0.01 in wild type and 0.652 ± 0.016 in tdp1-deficient mutant. Nearly 46% of DPCs were repaired in wild type cells 6 hours after the formaldehyde treatment, while only 35% of DPCs were repaired in *tdp1* cells. Asterisks (*) indicate p < 0.05 by the Student’s *t*-test; (B, C) Formaldehyde did not form trapped TOPO1. TOPO1 was detected by western blotting (B) and slot blotting (C). Trapped TOPO1 was not detected in chromosomal DNA after the treatment with formaldehyde; (D) The formation of DPCs containing histone H3 was detected in the genome, demonstrating DPC formation by formaldehyde.

We utilized a FITC-labeling method to measure the amount of DPCs in genome DNA. Cells were treated with 0.2 mM formaldehyde for 3 hours and chromosomal DNA was purified. DPCs in purified chromosomal DNA was labeled with FITC and the amount of DPCs were quantified by fluorometry. The fluorescence intensities (arbitrary unit) of DNA samples from the formaldehyde-treated cells (130.6 ± 7.26) was significantly higher than the one from the control cells (20.3 ± 1.95) (S2 Fig). The results confirm that formaldehyde generates DPCs. The fluorescence intensities of the control untreated cells with and without the proteinase K-treatment were 6.4 ± 0.59 and 20.3 ± 1.95, respectively (S2 Fig). This difference in fluorescence intensity (13.9 = 20.3–6.4) was attributed to endogenous DPCs. The remainder (6.4 ± 0.59) was the background due to the non-specific reaction of FITC with DNA. To study the kinetics of the removal of DPCs, DT40 cells were incubated in the presence of 0.2 mM formaldehyde for 3 hours and then further incubated in the absence of formaldehyde for 0, 3, and 6 hours. Chromosomal DNA was isolated and analyzed for DPCs [26]. As shown in Fig 3A, the FITC signal was decreased during the post-incubation in wild type cells. The rate of the removal of DPCs was slower in tdp1-deficient cells than in wild type cells (Fig 3A). These results suggest that TDP1 plays a role in removing formaldehyde-induced DPCs. The remaining removal activity of DPCs in tdp1-deficient cells also suggests the existence of a TDP1-independent pathway to remove formaldehyde-induced DPCs.

A well-established target of TDP1 is trapped TOPO1. Therefore, the results in Fig 2A may be related to trapped TOPO1. To investigate whether formaldehyde induces trapped TOPO1 in DNA, chromosomal DNA from formaldehyde-treated cells was purified [26]. The purified DNA containing covalently linked proteins was digested with DNase I, and the resulting products were separated by SDS-polyacrylamide gel electrophoresis. TOPO1 was detected by Western blotting (Fig 3B) and Slot blotting (Fig 3C). The same sample was also separated and analyzed for histone H3 DPCs by Western blots as a positive control (Fig 3D). Trapped TOPO1 was not observed in formaldehyde-treated cells (Fig 3B and 3C), while formaldehyde induced histone H3 DPCs in both wild type and *tdp1* cells (Fig 3D). To verify our experimental system for the detection of trapped TOPO1, we treated cells with 0.1 μM CPT and analyzed the formation of trapped TOPO1. The concentration of CPT was selected to give a similar level of cell survival that was induced by 0.2 mM formaldehyde. Trapped TOPO1 was clearly detected in CPT-treated cells under the conditions used (S4 Fig). We concluded that formaldehyde did not induce DPCs containing TOPO1, and that the sensitivity to formaldehyde observed in *tdp1* cells was not due to trapped TOPO1, the primary substrate for TDP1.

Prasad et al. recently reported that DPCs containing PARP1 is a substrate of TDP1 [58]. This finding raises the possibility that PARP1-DPCs are produced by formaldehyde and are the cytotoxic lesion. If PARP1-DPCs are a primary cytotoxic lesion, the *tdp1*/*parp1* cells will show less cytotoxicity compared to the *tdp1* cells due to lack of the formation of PARP1-DPCs. Fig 2B demonstrates that a deletion of PARP1 in the *tdp1* cells does not alter the sensitivity to formaldehyde. Hence, PARP1-DPCs are not likely a primary cytotoxic lesion.

### Accumulation of chromosomal aberrations in tdp1-deficient cells by formaldehyde

**Fig 4:**
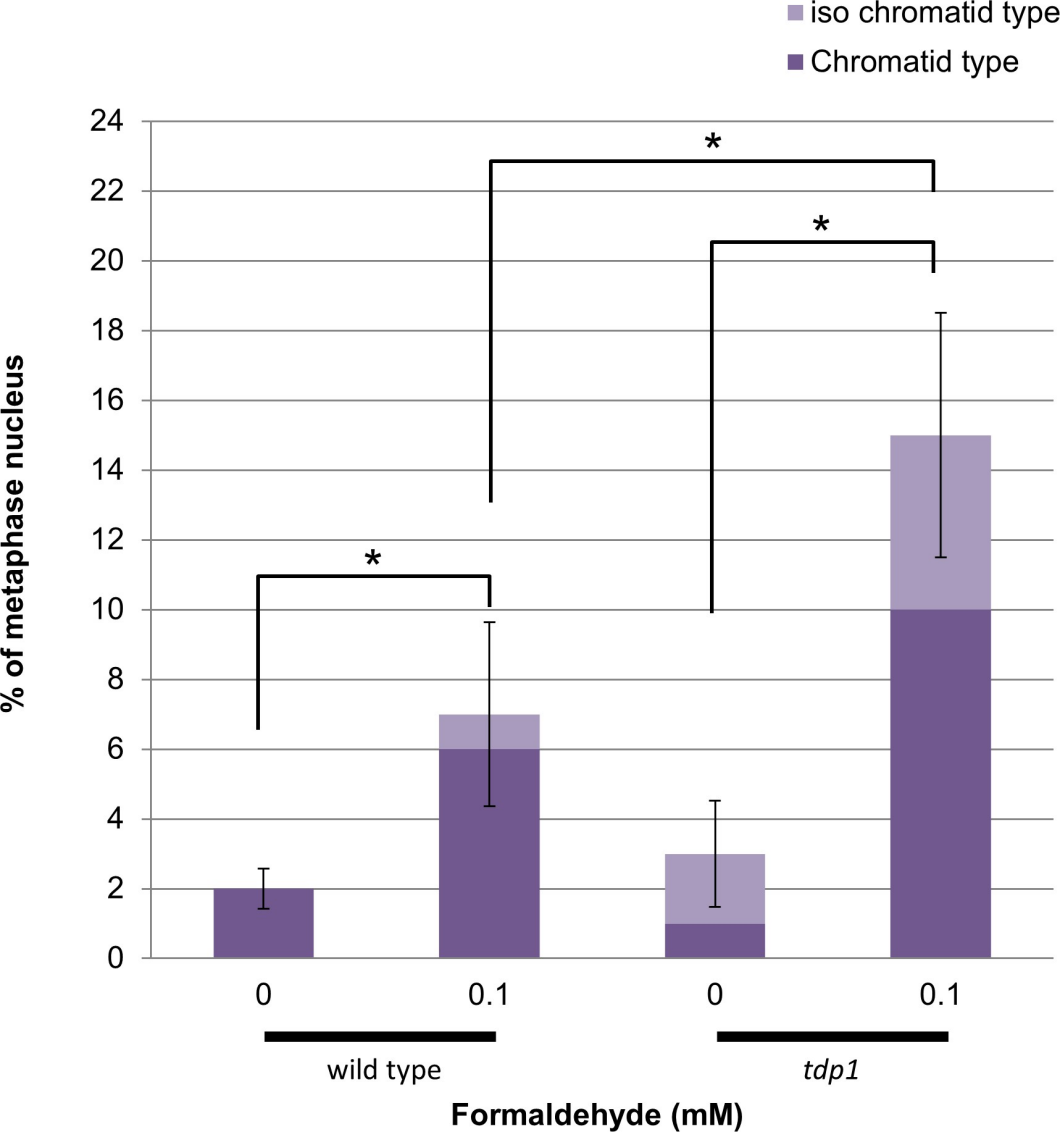
Elevated levels of chromosomal aberrations induced by formaldehyde in *tdp1* cells. Cells were treated with formaldehyde and the numbers of chromatid and iso-chromatid breaks were counted per 20 metaphase nuclei. The chromatid and iso-chromatid types of chromosomal aberrations were both elevated in the absence of TDP1. All data represent the mean ± SD of three independent experiments. Asterisks (*) indicate p < 0.05 by a multiple-comparison one-way ANOVA (Tukey’s test).

Previous studies showed that highly toxic aldehydes, such as alpha-beta unsaturated aldehyde, directly attack proteins rather than DNA and kill cells [59]. In contrast, cytotoxicity induced by formaldehyde is caused by DNA damage [59]. Based on evidence for the existence of the TDP1-mediated repair of formaldehyde-induced DPCs, we investigated the genotoxicity of formaldehyde. We analyzed chromosomal aberrations as direct evidence of DNA damage in mitotic chromosomes after the exposure of wild type and *tdp1* cells to formaldehyde. We were particularly interested in chromatid- and iso-chromatid-type breaks (S5 Fig). Iso-chromatid breaks are generated through DSBs that are induced before and during DNA replication [45, 60]. Chromatid breaks are formed through DSBs that are introduced after DNA replication [45, 60]. As shown in Fig 4, formaldehyde induced both types of chromosomal breakages more efficiently in *tdp1* cells than in wild type cells, suggesting DSBs are generated by formaldehyde-induced DPCs. Moreover, a percentage of cells with iso-chromatid breaks was 1% in wild type cells and 5% in tdp1-deficient cells, while a percentage of cells with chromatid breaks was 6% in wild type cells and 10% in tdp1-deficient cells. These data indicate that formaldehyde-induced DPCs may induce DSBs during DNA replication. Thus, the blockage of the DNA replication machinery by the accumulated formaldehyde-induced DPCs and or an abortive DNA repair process in the absence of TDP1 resulted in chromosomal breakages that lead to cell death.

The mechanism of TDP1-mediated repair of DPCs induced by formaldehyde is unclear. TDP1 acts on the 3’-blocked end at a strand break and removes the 3’-blocked end by 3’-cleavage activity [36–38]. Thus, a 3’-end proximal to DPC may be required for the DPC repair activity of TDP1. Since formaldehyde introduces DPCs without the disruption of the DNA strand, it is unlikely that the 3’-end blocking DNA lesions that are the canonical substrate for TDP1, are generated by formaldehyde. Induction of chromosomal breaks by formaldehyde shown in Fig 4 suggests that DSBs might be generated during DNA replication and as an abortive DNA repair process. These DSBs can serve as a substrate for TDP1. Other endonucleases such as MRE11 may directly make an incision at close proximity to the 3’-side of DPC and generate a substrate for TDP1 to remove the DPC. Alternatively, TDP1 may function as a scaffold protein without requiring the 3’-cleavage activity by mediating protein-protein interactions during the removal of DPCs.

In summary, we herein provided evidence that TDP1 mediates the repair of formaldehyde-induced DPCs that are distinct from trapped TOPO1. Further studies are needed to identify the formaldehyde-induced cytotoxic DNA lesions repaired by TDP1 and decipher the mechanism of TDP1-mediated DPC repair.

## Supporting information

S1 Table DT40 cells defective in Fanconi anemia repair pathway cell lines used in this study. (DOCX)

S1 Fig Structures of three types of DPCs. Pink-colored pentagons indicate topoisomerase inhibitors in type 2 and type 3 DPCs. “P” indicates the 3’- or 5’-terminal phosphate group cross-linked to topoisomerases.
(TIF)

S2 Fig Assessment of the amount of endogenous DPCs in DT40 cells by the FITC-fluorescent labeling method. Protein fractions in DPCs were labeled with 0.1 mM FITC. The fluorescence intensities of DNA from control cells without a drug treatment with or without the proteinase K-treatment were 6.4 ± 0.59 and 20.3 ± 1.95, respectively. The difference observed in fluorescence intensity (13.9 = 20.3–6.4) was attributed to endogenous DPCs. The remainder (6.4) was the background due to the non-specific binding of FITC with DNA. The fluorescence intensities of DNA from cells that were incubated with 0.2 mM formaldehyde for 3 hours with or without the proteinase K-treatment were 6.8 ± 0.25 and 103.6 ± 7.26, respectively.
(TIF)

S3 Fig TDP1 and Fanconi anemia pathway-related proteins involved in the repair of formaldehyde- and MMC-induced DNA lesions. (A) Tdp1-, tdp2-deficient cells are proficient in ICL repair. MMC was not toxic to *tdp1* or *tdp2* cells; (B) Fancd2- and fancc-deficient cells are defective in ICL repair. *Fancd2* and *fancc* cells were hypersensitive to MMC. All data in (A) and (B) represent the means ± SD of three independent experiments; (C, D) Histograms of the IC_50_ values of formaldehyde (C) and MMC (D) in wild type and cells deficient in Fanconi anemia-related proteins and TDP1. Cells were treated with formaldehyde for 3 hours or MMC for 24 hours and colonies formed on complete media. All data represent IC_50_ of 95% confidence intervals. Formaldehyde was more cytotoxic in Fanconi anemia-deficient cells than in *tdp1* cells. This additional sensitivity to formaldehyde in Fanconi anemia mutants could be due to the concurrent formation of ICLs and DPCs. and also implies that the Fanconi anemia pathway is required in both ICL and DPC repair.
(TIF)

S4 Fig Detection of trapped TOPO1 in chromosomal DNA after the treatment with CPT. Cells were treated with formaldehyde or CPT for 3 hours at the indicated concentrations. After removing the media containing CPT, chromosomal DNA was isolated by two rounds of the CsCl gradient, and trapped TOPO1 was detected by Western blotting. Formaldehyde did not induce trapped TOPO1 while CPT efficiently trapped TOPO1. Purified TOPO1 (topo1) was included as a positive control.
(TIF)

S5 Fig Representative images of chromatid and iso-chromatid breaks. Images were taken from the wild type DT40 cells were exposed to MMC at 20 ng/ml for 16 hours. The arrows indicate chromatid break in the left image and iso-chromatid break in the right image.
(TIF)

S1 Raw Images (PDF)
