# Supplementary material for: Participation of TDP1 in the repair of formaldehyde-induced DNA-protein cross-links in chicken DT40 cells
Source: PLoS One. 2020 Jun 26;15(6):e0234859. doi: 10.1371/journal.pone.0234859 (PMC7319324; doi:10.1371/journal.pone.0234859)
Supplement: S1 Table — (DOCX) [file pone.0234859.s001.docx]

| **Cell line** | **Function inactivated** | **References** |
| --- | --- | --- |
| *tdp1* | SSB and DSB repair of the trapped TOPO1 and TOPO2 | (1) |
| *tdp2* | Removal of the trapped TOPO2 | (2) |
| *tdp1, tdp2* | Removal of the trapped TOPO1 and the trapped TOPO2 | (3) |
| *parp-1* | DNA damage sensing, poly(ADP-rybosyl)ation | (4) |
| *sprtn* | DNA damage tolerance | (5) |
| *fancc* | Fanconi anemia core complex | (6) |
| *fancd1* | Homologous recombination and fork stabilization | (7) |
| *fancd2* | Binds to FANCI; has multiple functions | (8) |
| *fance* | Fanconi anemia core complex | (9) |
| *fancg* | Fanconi anemia core complex | (10) |
| *fanci* | Binds to FANCD2; has multiple functions | (11) |
| *fancj* | Homologous recombination and translesion synthesis | (12) |
| *fancl* | Fanconi anemia core complex; E3 ubiquitin ligase for FNACD2-I ubiquitylation | (13) |
| *fancm* | Fanconi anemia core complex; lesion recognition; landing platform for the Fanconi anemia core complex | (14) |
| *faap100* | Fanconi anemia core complex | (15) |

**References**

1. Murai J, Huang SY, Das BB, Dexheimer TS, Takeda S, Pommier Y. Tyrosyl-DNA phosphodiesterase 1 (TDP1) repairs DNA damage induced by topoisomerases I and II and base alkylation in vertebrate cells. J Biol Chem. 2012;287: 12848–12857.
2. Zeng Z, Cortes-Ledesma F, El Khamisy SF, Caldecott KW. TDP2/TTRAP is the major 5'-tyrosyl DNA phosphodiesterase activity in vertebrate cells and is critical for cellular resistance to topoisomerase II-induced DNA damage. J Biol Chem. 2011;286: 403–409.
3. Zeng Z, Sharma A, Ju L, Murai J, Umans L, Vermeire L, et al. TDP2 promotes repair of topoisomerase I-mediated DNA damage in the absence of TDP1. Nucleic Acids Res. 2012;40: 8371–8380.
4. Hochegger H, Dejsuphong D, Fukushima T, Morrison C, Sonoda E, Schreiber V, et al. Parp-1 protects homologous recombination from interference by Ku and Ligase IV in vertebrate cells. EMBO J. 2006;25: 1305–1314.
5. Nakazato A, Kajita K, Ooka M, Akagawa R, Abe T, Takeda S, et al. SPARTAN promotes genetic diversification of the immunoglobulin-variable gene locus in avian DT40 cells. DNA Repair (Amst). 2018; 68: 50–57.
6. Hirano S, Yamamoto K, Ishiai M, Yamazoe M, Seki M, Matsushita N, et al. Functional relationships of FANCC to homologous recombination, translesion synthesis, and BLM. EMBO J. 2005;24: 418–427.
7. Qing Y, Yamazoe M, Hirota K, Dejsuphong D, Sakai W, Yamamoto KN, et al. The epistatic relationship between BRCA2 and the other RAD51 mediators in homologous recombination. PLoS Genet. 2011;7: e1002148. doi: 10.1371/journal.pgen.1002148
8. Yamamoto K, Hirano S, Ishiai M, Morishima K, Kitao H, Namikoshi K, et al. Fanconi anemia protein FANCD2 promotes immunoglobulin gene conversion and DNA repair through a mechanism related to homologous recombination. Mol Cell Biol. 2005;25: 34–43.
9. Huang Y, Leung JW, Lowery M, Matsushita N, Wang Y, Shen X, et al. Modularized functions of the Fanconi anemia core complex. Cell Rep. 2014;7: 1849–1857.
10. Yamamoto K, Ishiai M, Matsushita N, Arakawa H, Lamerdin JE, Buerstedde JM, et al. Fanconi anemia FANCG protein in mitigating radiation- and enzyme-induced DNA double-strand breaks by homologous recombination in vertebrate cells. Mol Cell Biol. 2003;23:5421–5430.
11. Ishiai M, Kitao H, Smogorzewska A, Tomida J, Kinomura A, Uchida E, et al. FANCI phosphorylation functions as a molecular switch to turn on the Fanconi anemia pathway. Nat Struct Mol Biol. 2008;15: 1138–1146.
12. Wu Y, Sommers JA, Suhasini AN, Leonard T, Deakyne JS, Mazin AV, er al. Fanconi anemia group J mutation abolishes its DNA repair function by uncoupling DNA translocation from helicase activity or disruption of protein-DNA complexes. Blood. 2010 ;116: 3780–3791.
13. Seki S, Ohzeki M, Uchida A, Hirano S, Matsushita N, Kitao H, et al. A requirement of FancL and FancD2 monoubiquitination in DNA repair. Genes Cells. 2007;12: 299–310.
14. Ling C, Huang J, Yan Z, Li Y, Ohzeki M, Ishiai M, et al. Bloom syndrome complex promotes FANCM recruitment to stalled replication forks and facilitates both repair and traverse of DNA interstrand crosslinks. Cell Discov. 2016;2:16047. doi:10.1038/celldisc.2016.47
15. Ling C, Ishiai M, Ali AM, Medhurst AL, Neveling K, Kalb R, et al. FAAP100 is essential for activation of the Fanconi anemia-associated DNA damage response pathway. EMBO J. 2007;26: 2104–2114.
