# Supplementary figures and images for: Participation of TDP1 in the repair of formaldehyde-induced DNA-protein cross-links in chicken DT40 cells
Source: PLoS One. 2020 Jun 26;15(6):e0234859. doi: 10.1371/journal.pone.0234859 (PMC7319324; doi:10.1371/journal.pone.0234859)

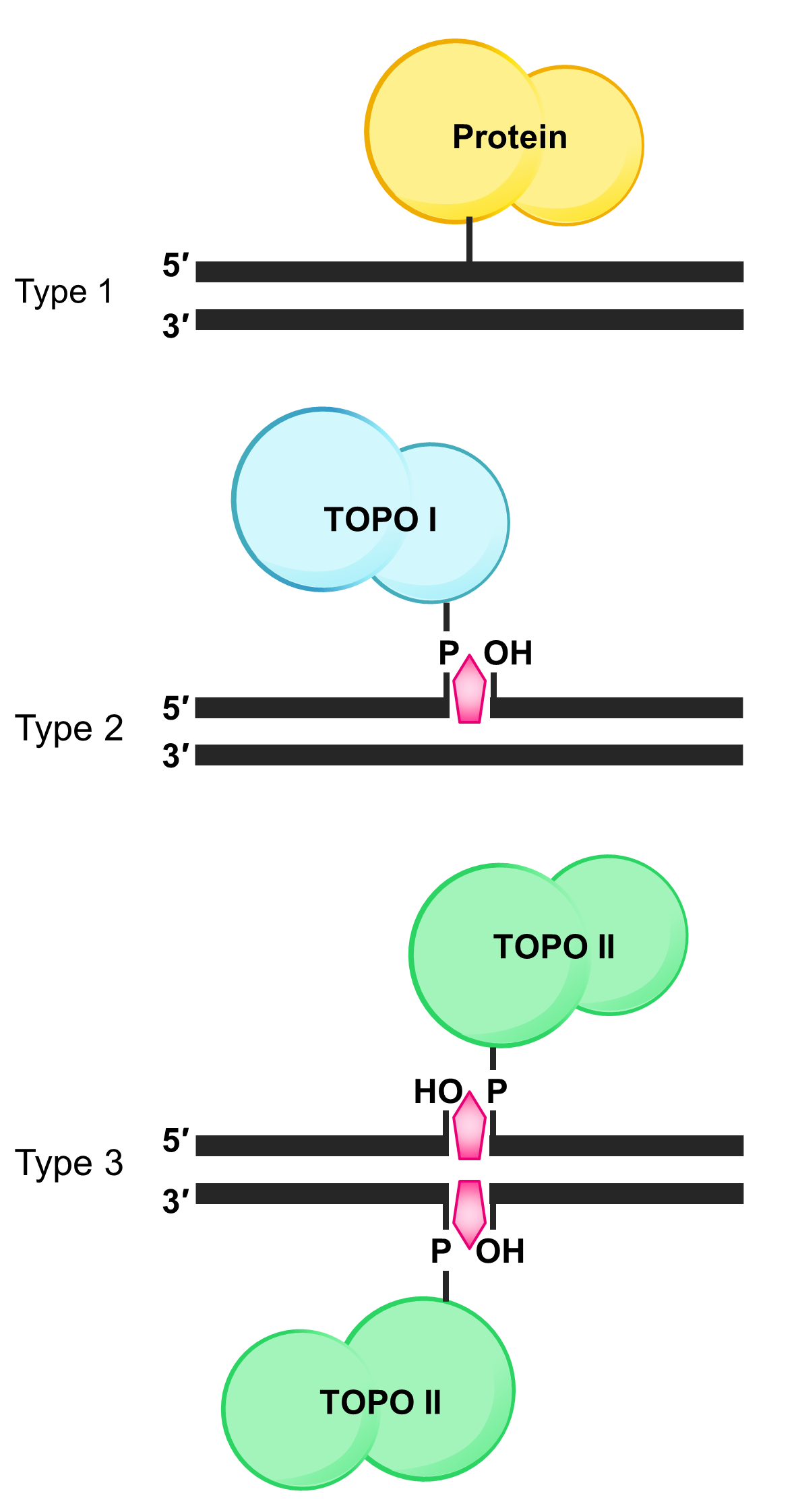

Supplement: S1 Fig — Pink-colored pentagons indicate topoisomerase inhibitors in type 2 and type 3 DPCs. “P” indicates the 3’- or 5’-terminal phosphate group cross-linked to topoisomerases. (TIF) [file pone.0234859.s002.tif]

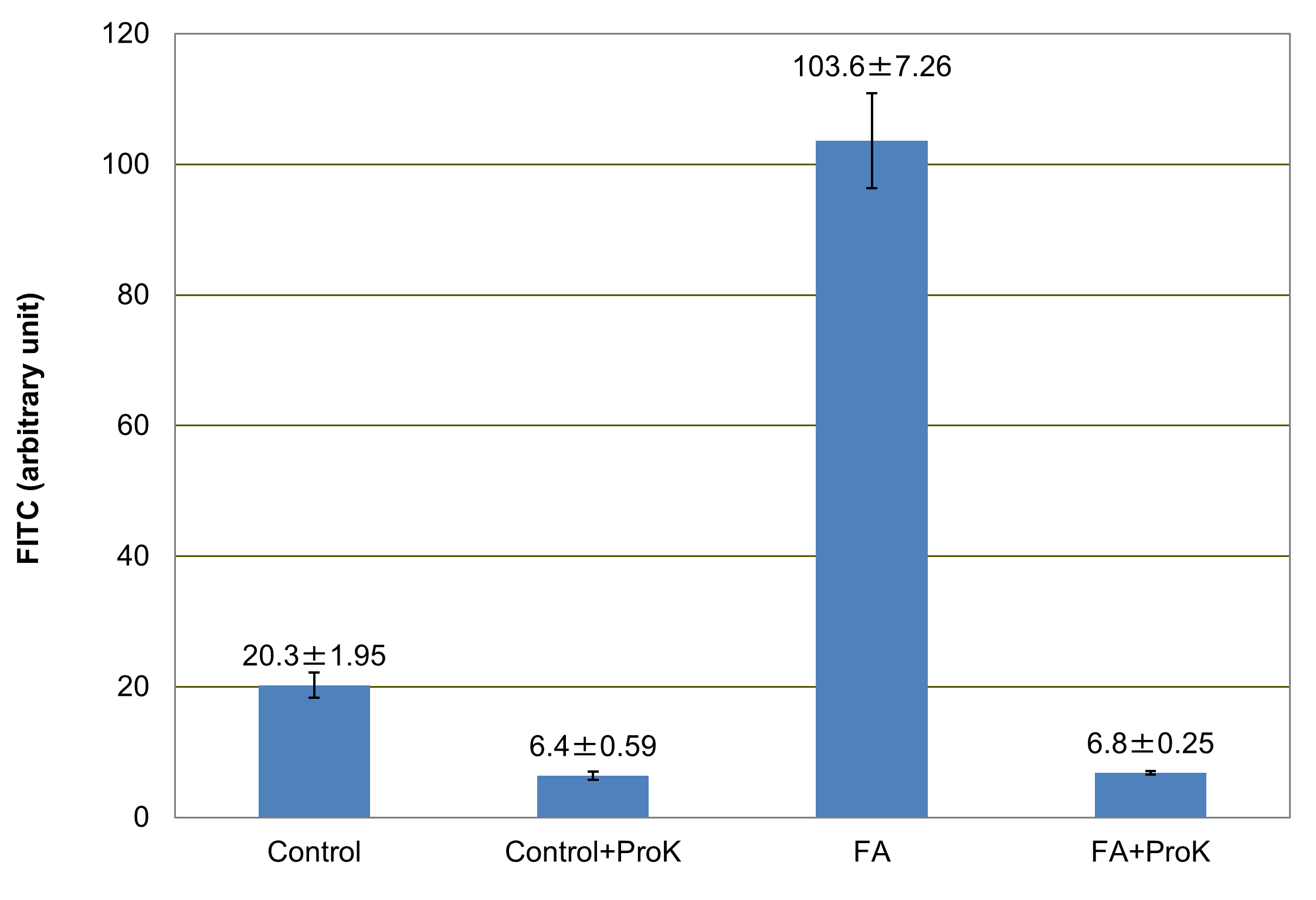

Supplement: S2 Fig — Protein fractions in DPCs were labeled with 0.1 mM FITC. The fluorescence intensities of DNA from control cells without a drug treatment with or without the proteinase K-treatment were 6.4 ± 0.59 and 20.3 ± 1.95, respectively. The difference observed in fluorescence intensity (13.9 = 20.3–6.4) was attributed to endogenous DPCs. The remainder (6.4) was the background due to the non-specific binding of FITC with DNA. The fluorescence intensities of DNA from cells that were incubated with 0.2 mM formaldehyde for 3 hours with or without the proteinase K-treatment were 6.8 ± 0.25 and 103.6 ± 7.26, respectively. (TIF) [file pone.0234859.s003.tif]

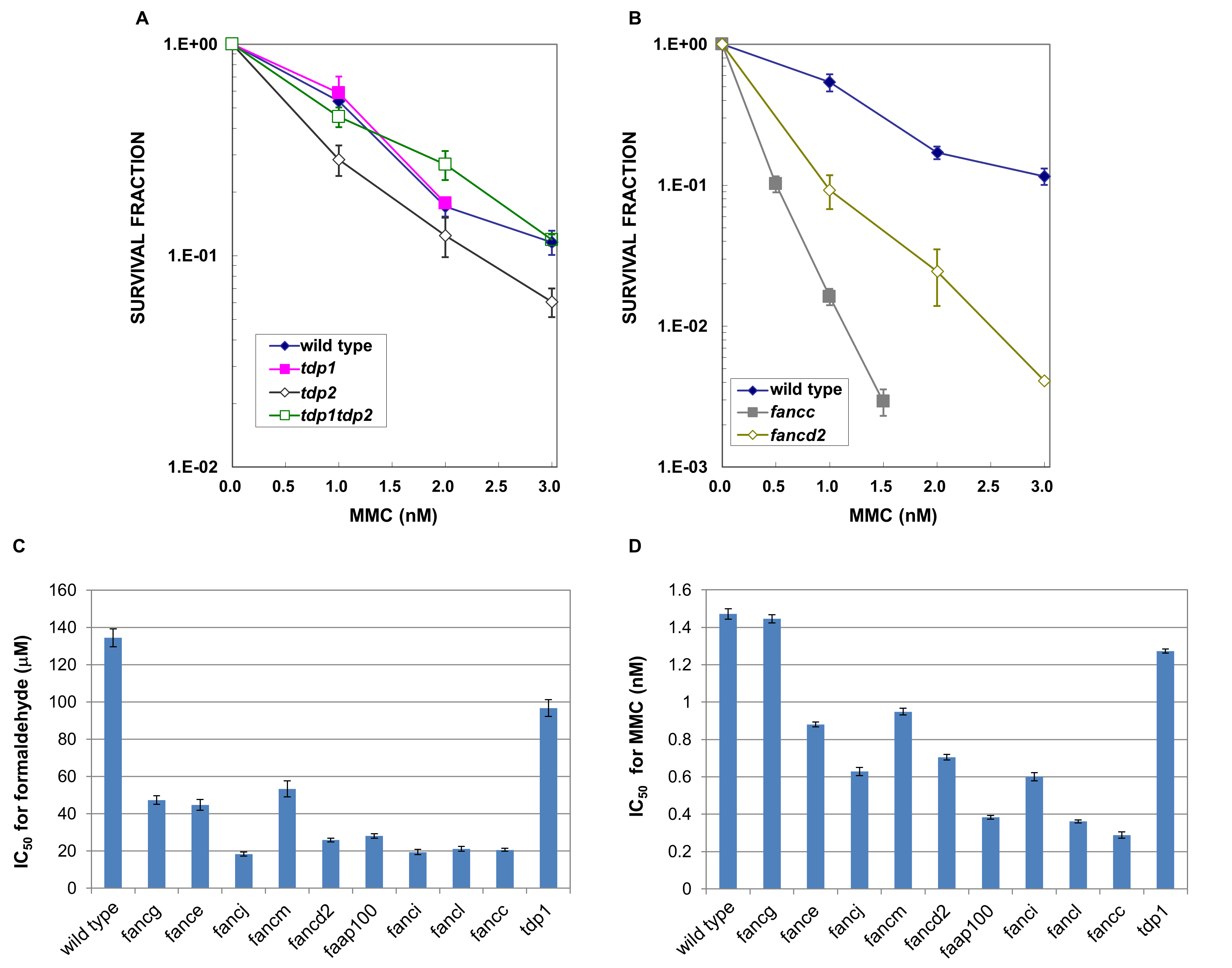

Supplement: S3 Fig — (A) Tdp1-, tdp2-deficient cells are proficient in ICL repair. MMC was not toxic to tdp1 or tdp2 cells; (B) Fancd2- and fancc-deficient cells are defective in ICL repair. Fancd2 and fancc cells were hypersensitive to MMC. All data in (A) and (B) represent the means ± SD of three independent experiments; (C, D) Histograms of the IC50 values of formaldehyde (C) and MMC (D) in wild type and cells deficient in Fanconi anemia-related proteins and TDP1. Cells were treated with formaldehyde for 3 hours or MMC for 24 hours and colonies formed on complete media. All data represent IC50 of 95% confidence intervals. Formaldehyde was more cytotoxic in Fanconi anemia-deficient cells than in tdp1 cells. This additional sensitivity to formaldehyde in Fanconi anemia mutants could be due to the concurrent formation of ICLs and DPCs. and also implies that the Fanconi anemia pathway is required in both ICL and DPC repair. (TIF) [file pone.0234859.s004.tif]

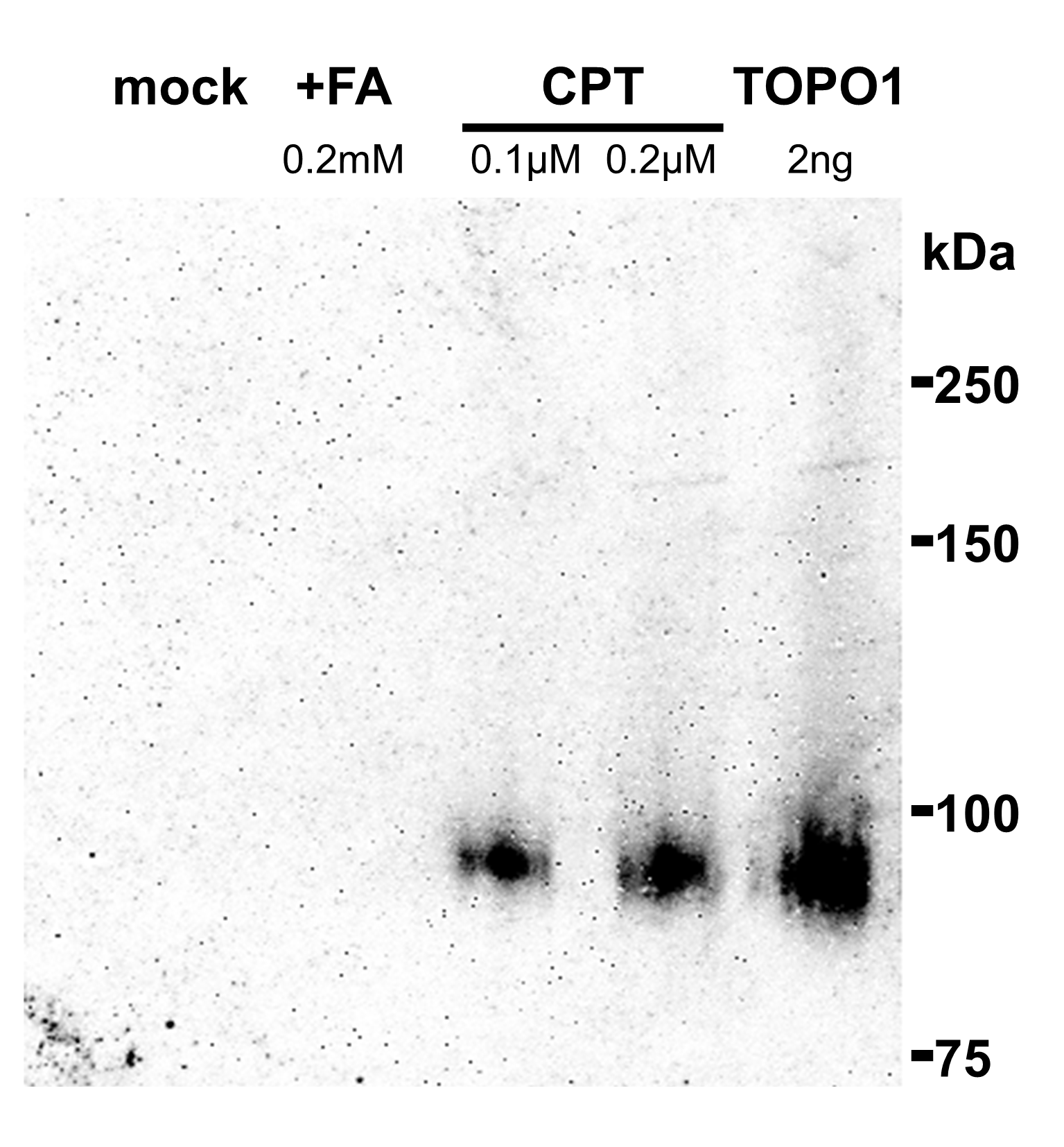

Supplement: S4 Fig — Cells were treated with formaldehyde or CPT for 3 hours at the indicated concentrations. After removing the media containing CPT, chromosomal DNA was isolated by two rounds of the CsCl gradient, and trapped TOPO1 was detected by Western blotting. Formaldehyde did not induce trapped TOPO1 while CPT efficiently trapped TOPO1. Purified TOPO1 (topo1) was included as a positive control. (TIF) [file pone.0234859.s005.tif]

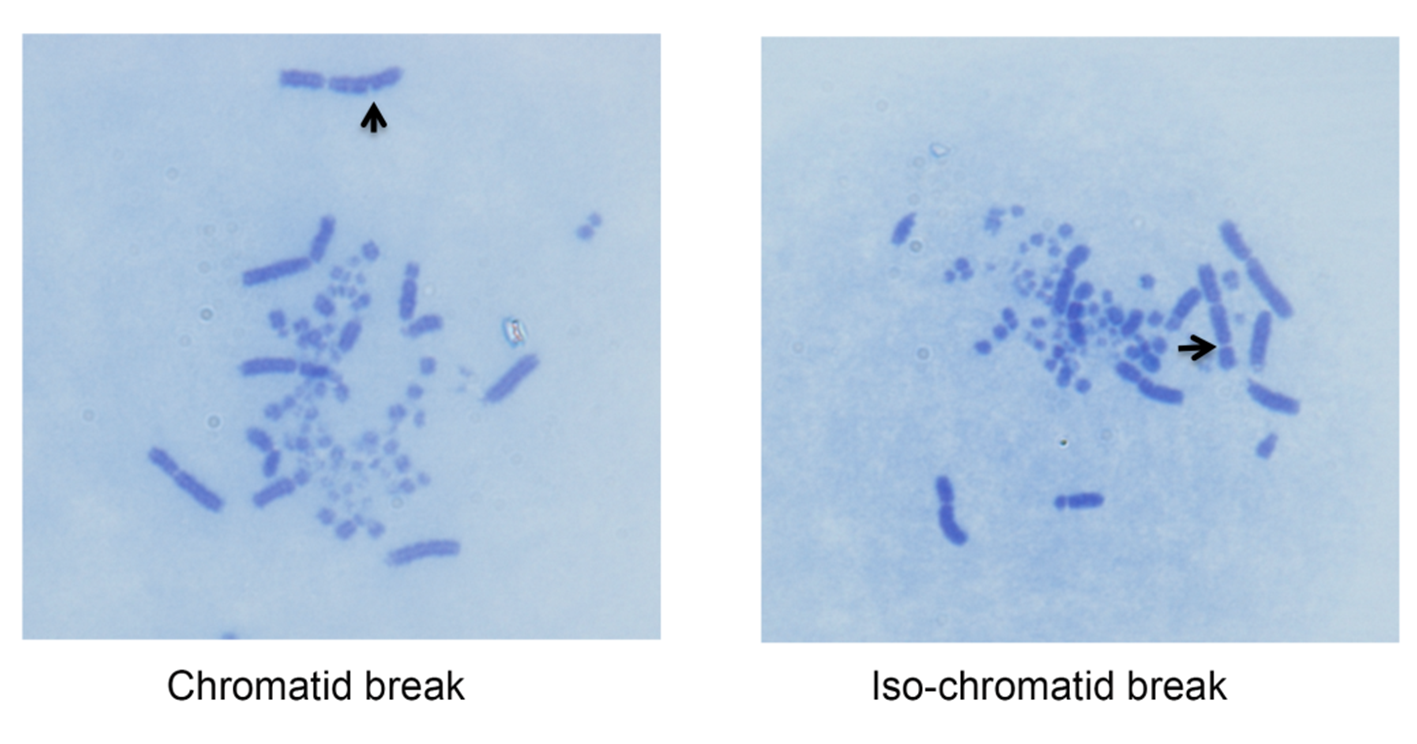

Supplement: S5 Fig — Images were taken from the wild type DT40 cells were exposed to MMC at 20 ng/ml for 16 hours. The arrows indicate chromatid break in the left image and iso-chromatid break in the right image. (TIF) [file pone.0234859.s006.tif]
