# Supplementary material for: Participation of TDP1 in the repair of formaldehyde-induced DNA-protein cross-links in chicken DT40 cells
Source: PLoS One. 2020 Jun 26;15(6):e0234859. doi: 10.1371/journal.pone.0234859 (PMC7319324; doi:10.1371/journal.pone.0234859)

S1_raw_images

Below is the raw image for Fig. 3B. After processing the conversion black and white to generate white version, and the adjustment contrast and brightness without any arbitrary manipulation by ImageJ, the image was cropped to boxed area except the two lanes marked with a red "X." Each molecular size is based on the non-fluorescent pre-stained size marker in Fig. 3B.

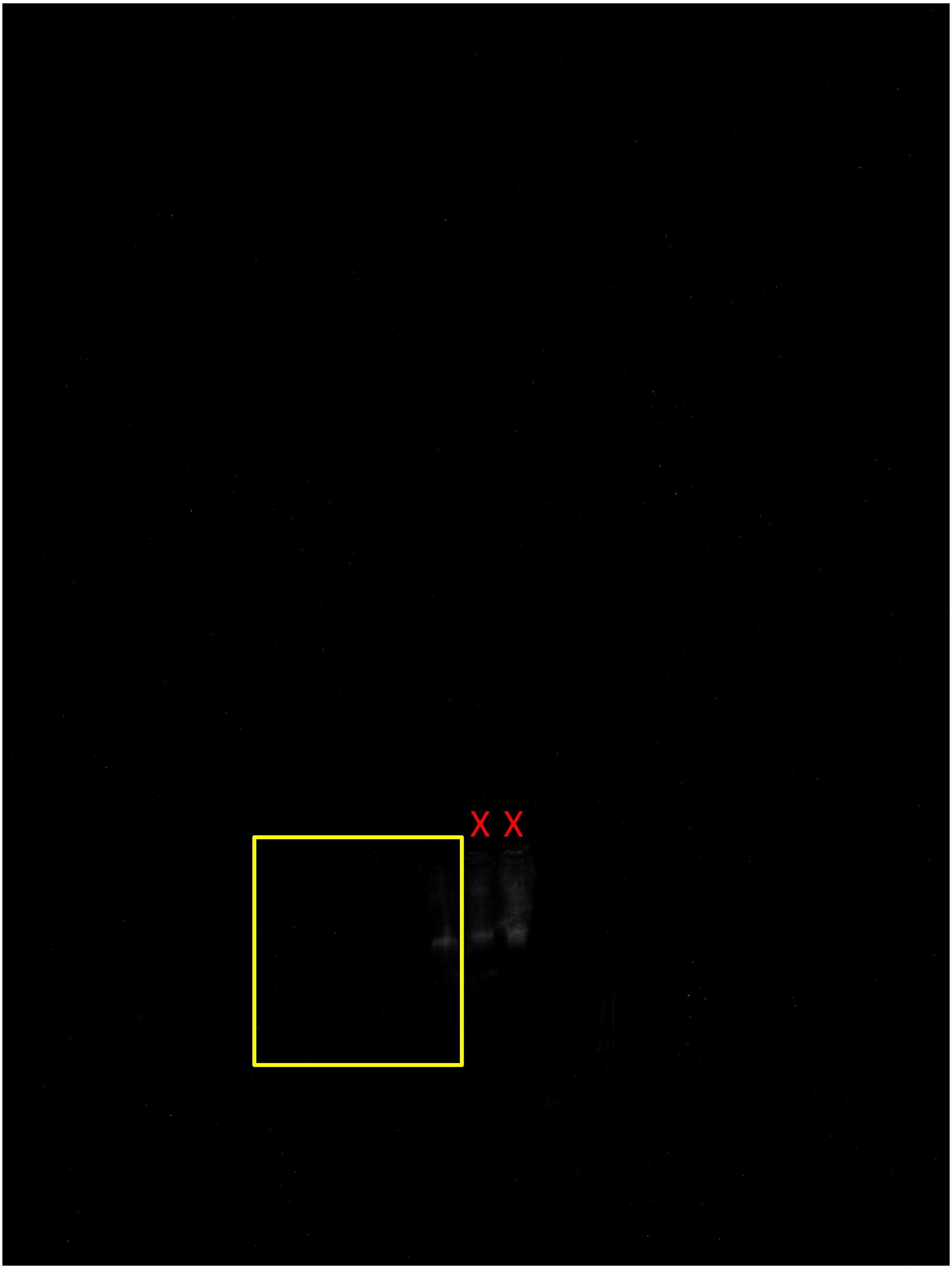

Below is the raw image for Fig. 3C. After processing the conversion black and white to generate white version, and the adjustment contrast and brightness without any arbitrary manipulation by ImageJ, the image was cropped to boxed area.

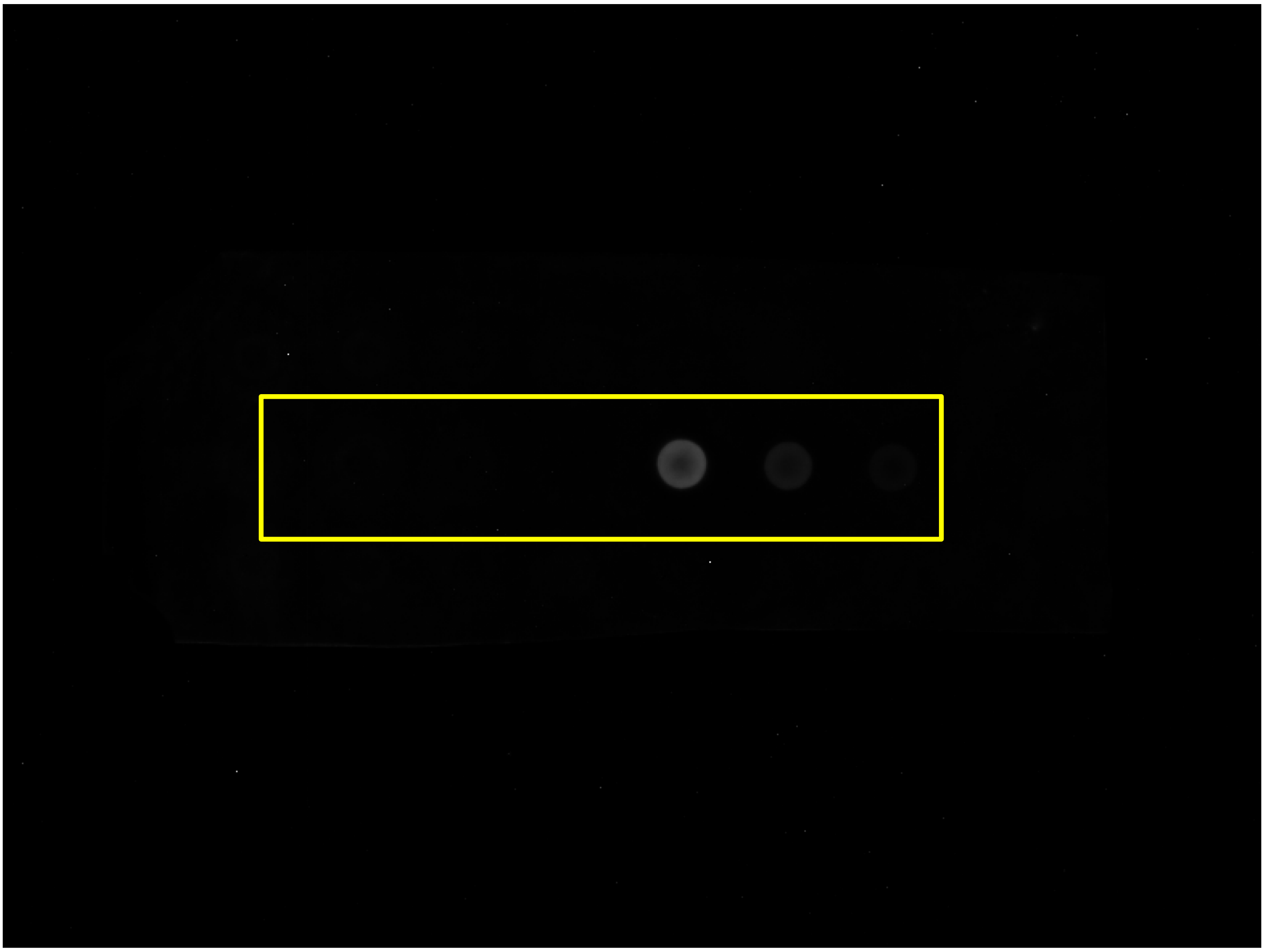

Below is the raw image for Fig. 3D. After processing the adjustment contrast and brightness without any arbitrary manipulation by ImageJ, the image was cropped to boxed area except the lane marked with a red “X” and molecular size marker.

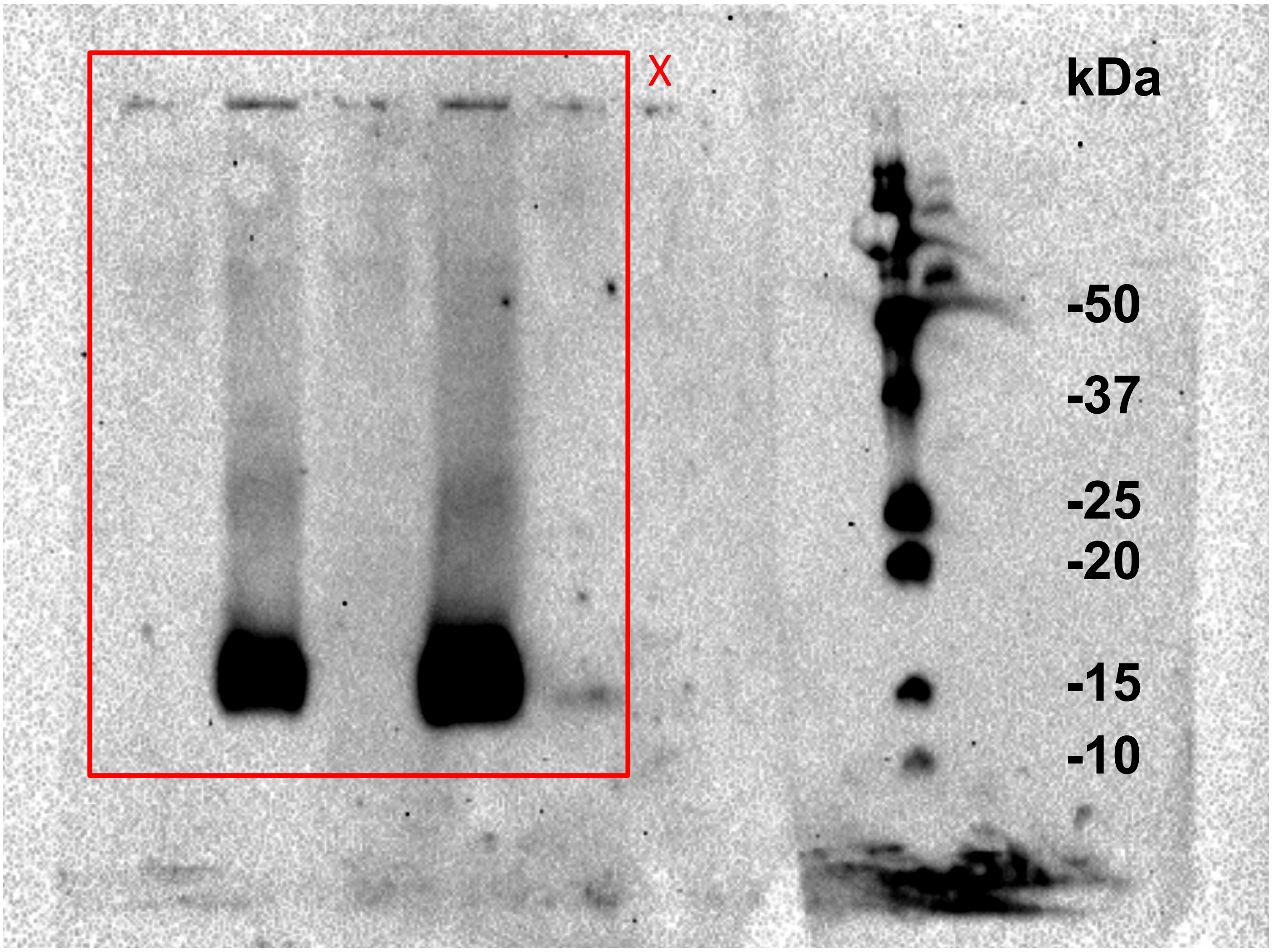

Below is the raw image for S4 Fig. After processing the conversion black and white to generate white version, and the adjustment contrast and brightness without any arbitrary manipulation by ImageJ, the image was cropped to boxed area except the three lanes marked with a red "X." Each molecular size is based on the non-fluorescent pre-stained size marker in S4 Fig.

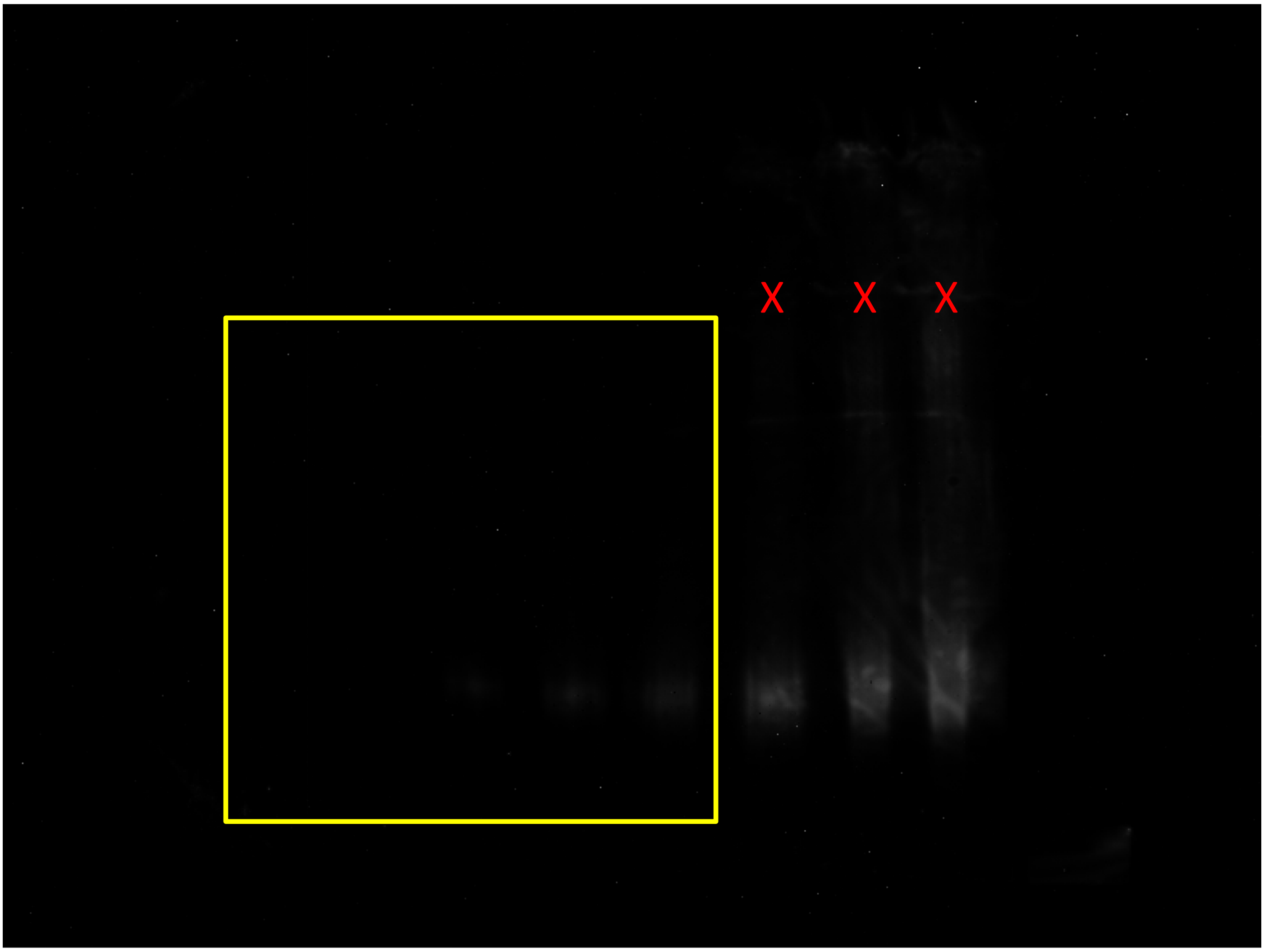

Supplement: S1 Raw Images — (PDF) [file pone.0234859.s007.pdf]
